# Adverse Childhood Experiences and Adult Smoking, Nebraska, 2011

**DOI:** 10.5888/pcd10.130009

**Published:** 2013-09-19

**Authors:** Kristin Yeoman, Thomas Safranek, Bryan Buss, Betsy L. Cadwell, David Mannino

**Affiliations:** Author Affiliations: Thomas Safranek, Nebraska Department of Health and Human Services, Lincoln, Nebraska; Bryan Buss, Betsy L. Cadwell, Centers for Disease Control and Prevention, Atlanta, Georgia; David Mannino, University of Kentucky College of Public Health, Lexington, Kentucky.

## Abstract

**Introduction:**

Smoking is a public health risk; the prevalence of smoking among adults in Nebraska is 18.4%. Studies indicate that maltreatment of children alters their brain development, possibly increasing risk for tobacco use. Previous studies have documented associations between childhood maltreatment and adult health behaviors, demonstrating the influence of adverse experiences on tobacco use. We examined prevalence and associations between adverse childhood experiences and smoking among Nebraskans.

**Methods:**

We analyzed 2011 Nebraska Behavioral Risk Factor Surveillance System (Adverse Childhood Experience module) data, defining adverse childhood experience exposures as physical, sexual, and verbal abuse (ie, direct exposures), and household dysfunction associated with mental illness, substance abuse, divorce, domestic violence, and living with persons with incarceration histories (ie, environmental exposures). We estimated prevalence of exposures, taking into account the complex survey design. We used logistic regression with predicted margins to estimate adjusted relative risk for smoking by direct or environmental exposure.

**Results:**

Approximately 51% of Nebraskans experienced 1 or more adverse childhood events; 7% experienced 5 or more. Prevalence of environmental exposures (42%) was significantly higher than that of direct exposures (31%). Prevalence of individual exposures ranged from 6% (incarceration of a household member) to 25% (verbal abuse). Adjusted relative risks of smoking for direct and environmental exposures were 1.5 and 1.8, respectively.

**Conclusion:**

We present a new method of evaluating adverse childhood experience data. Prevalence of adverse childhood experiences is high among Nebraskans, and these exposures are associated with smoking. State-specific strategies to monitor adverse events among children and provide interventions might help to decrease the smoking rate in this population.

## Introduction

Tobacco use is the leading underlying cause of death in the United States (1) and likewise is an important public health problem in Nebraska. During 2010, prevalence of adult smoking in Nebraska was 18.4%, similar to the national average of 19.0% (2,3). Preventing or delaying the onset of tobacco use is a key component in decreasing the occurrence of premature deaths. Understanding the genetic and environmental factors that predispose people to tobacco use is important. One area of study that has received substantial interest involves childhood adverse events. Experiencing childhood sexual and physical abuse and witnessing violence in the home or community substantially increase the risk of tobacco initiation and dependence (4–6).

Investigators are beginning to elucidate the underlying mechanisms by which abuse affects childhood development and subsequent pathology. Changes in hippocampal volume have been demonstrated among people affected by childhood trauma (7). Dysregulation of stress responses among victims of childhood trauma is postulated to increase their subsequent risk for substance abuse (8).

Life course theory (LCT) is an intergenerational approach to health and well-being and posits that health trajectories are developed during each person’s lifespan on the basis of genetic, economic, environmental, and social factors (9). LCT promotes broad-based strategies to focus resources on preventing adverse health determinants that occur earlier in the person’s life, developing integrated services across the lifespan and reducing risks and fostering protective factors at individual, family, and societal levels (9). Traditionally, public health and medicine have concentrated on disparities within individual disease states rather than on early health determinants. Adverse childhood experiences could be considered early determinants that contribute to adverse health trajectories through increased adoption of unhealthy behaviors.

The 1995 Adverse Childhood Experiences Study demonstrated the effect of childhood maltreatment and household dysfunction on adult smoking, obesity, and excessive alcohol use (10). During 2009, the Centers for Disease Control and Prevention (CDC) added an optional module to the core Behavioral Risk Factor Surveillance System (BRFSS) questions, adapting questions from the Adverse Childhood Experiences Study. That year, 5 states administered the Adverse Childhood Experiences module. An estimated 59% of residents in those states had 1 or more adverse childhood experience (ACE), and approximately 9% had 5 or more (11). Other studies have evaluated individual ACEs as primary exposures and compared outcomes among persons with and without individual ACE exposures; this method has the limitation of different referent groups for each individual ACE analysis, and thus no comparison can be made of the magnitude of associations between outcomes and individual ACEs. Findings from these earlier studies demonstrate a high prevalence of ACEs in a nonrandom selection of states. Establishing the prevalence of ACEs in each state can provide information to help the individual states target local prevention efforts.

Nebraska administered the ACE module during 2011, but the possible associations between ACEs and smoking among Nebraska residents have not been evaluated using these data. A limited number of divisions within Nebraska’s Department of Health and Human Services have expressed interest in using ACE data to guide data collection for new childhood maltreatment prevention and intervention programs. The behavioral health and children and family services divisions are focusing on prevention activities in addition to intervention strategies and can use results of state-specific ACE data analysis to assist in program development and education. The maternal and child health division initiated a home visitation program in 2012 and can use results of ACE data analysis to establish appropriate data requirements to monitor program outcomes. We undertook an investigation of ACE prevalence among Nebraska residents and its association with smoking, evaluating ACE data by using a new method adapted from a previous study (12).

## Methods

We analyzed data from the 2011 Nebraska BRFSS (13). BRFSS is a surveillance system administered by state health departments in conjunction with CDC. Each month, trained interviewers contacted a probability sample of adults in Nebraska to administer a core group of standardized questions regarding health behaviors and practices. A randomly selected subset of the total probability sample completed state-added optional modules, which included the ACE module, designed to obtain information of specific interest to Nebraska. Although the 2011 survey was the first to incorporate respondents with either landline or cellular telephone service, the ACE module did not include respondents with cellular telephones.

Response rates for BRFSS are calculated using standards set by the American Association of Public Opinion Research (AAPOR) Response Rate Formula #4 (www.aapor.org/Standard_Definitions2.htm). The response rate is the number of respondents who completed the survey as a proportion of all eligible and likely eligible persons. The response rate for Nebraska in 2011 was 66%. Detailed information is available in the BRFSS Summary Data Quality Report (14).

The ACE module comprised 11 questions regarding events that respondents experienced before age 18 years. We combined responses to 11 questions into the following ACE categories by adapting category definitions used in other studies as follows (10,11): physical abuse, sexual abuse, verbal abuse, mental illness in a household member, substance abuse in a household member, witnessing abuse, divorce, and incarceration of a household member. BRFSS questions and responses corresponding to individual ACE categories are listed in the Appendix.

We classified these 8 categories into direct and environmental ACEs. We defined direct ACEs as affirmative responses in any of the categories of physical abuse, sexual abuse, or verbal abuse. We defined environmental ACEs as affirmative responses in any of the categories of household mental illness, household substance abuse, witnessing abuse, divorce, or incarceration of a household member.

Definitions and age categories from the initial CDC BRFSS analyses were used to define control variables (11), but we categorized educational attainment into 4 categories (did not complete high school, completed high school, attended some college or technical school, or completed college or technical school), and we added excessive alcohol use as a covariate. We defined respondents as excessive alcohol users if they reported either heavy alcohol intake (≥60 or ≥30 alcoholic drinks for males or females, respectively, in 1 month) or binge drinking (≥5 or ≥4 alcoholic drinks for males or females, respectively, on a single occasion). Marital status and depression were evaluated but not included in the final model because they did not substantially affect the association of ACE status and smoking. The outcome variable of smoking was a self-reported smoker or never smoker. Only 5% (462) respondents were nonwhite, compared with the 2010 Census Bureau estimate for Nebraska of 13.9% nonwhite (15). Preliminary analyses of whites only and all races together reported no substantial differences. Therefore, because of the difficulty of interpreting results for multiple races together and the high probability of bias within the nonwhite sample, subsequent analyses were conducted only for non-Hispanic whites.

We included records with “yes” responses in any individual ACE category within the direct and environmental ACE groups, even in the presence of other missing responses, because inclusion in the direct and environmental groups required the presence of only 1 positive response in any individual ACE within that category. Missing responses in a record that contained “yes” responses would not modify the classification of that record as having a direct or environmental ACE, whereas missing responses in a record with other “no” responses could modify the classification. Therefore, we excluded from analysis records (7.4%) that were missing responses to all variables in either the direct or environmental ACE categories and records with only “no” and missing responses in the direct or environmental ACE categories.

We compared smoking among persons exposed to any of the 3 direct ACE variables with smoking among persons who did not report exposure to any ACE, among persons exposed to any of the 5 environmental ACEs with persons not reporting exposure to any ACE, and among persons exposed to both direct and environmental ACEs with persons not reporting any ACE exposure. We used SAS version 9.3 (SAS Institute Inc, Cary, North Carolina) and SUDAAN version 10.0.1 (RTI International, Research Triangle Park, North Carolina) to account for the complex survey design and weighting methodology used by BRFSS. We calculated prevalence estimates with 95% confidence intervals (CIs). We estimated adjusted relative risk (aRR) with 95% CIs of smoking by ACE status, controlling for age, sex, educational attainment, and excessive alcohol use, by using logistic regression models with predicted margins.

## Results

A total of 10,293 persons participated in the Nebraska BRFSS ACE module. Of these, 3,945 records (38%) were excluded because they were nonwhite, former smokers, or missing 1 or more exposures, control variables, or outcome variables. Of 6,348 remaining records, 49% had no ACEs; 33% had 1 or 2 ACEs; 11% had 3 or 4 ACEs; and 7% had 5 or more ACEs.

Prevalence of environmental ACEs (41.7%; 95% CI, 39.4−44.0) was significantly higher than that of direct ACEs (30.7%; 95% CI, 28.5−32.8) (Table 1). Prevalence of direct ACE exposures within age and education categories ranged from 26.1% (persons aged ≥55 years) to 36.0% (persons aged 25−34 years) and 28.7% (college graduates) to 33.2% (persons not completing high school), respectively. Prevalence of environmental ACE exposures within age and education categories ranged from 29.9% (persons aged ≥55 years) to 54.3% (persons aged 25−34 years) and 36.9% (college graduates) to 55.0% (persons not completing high school), respectively. Prevalence of individual ACEs ranged from 5.9% (household incarceration) to 25.1% (verbal abuse) (Table 2). Among persons with direct ACEs, 81.9% (95% CI, 79.1−84.7) experienced verbal abuse. Among persons with environmental ACEs, 56.7% (95% CI, 52.9−60.5) experienced household substance abuse.

**Table 1:** Prevalence of Direct^a^ and Environmental^b^ Adverse Childhood Events by Demographic Characteristic Among Non-Hispanic White Adults in Nebraska, Behavioral Risk Factor Surveillance System, 2011

Characteristic	Direct		Environmental	
	No. of Respondents	% (95% CI)	No. of Respondents	% (95% CI)
**Overall**	1,701	30.7 (28.5−32.8)	2,072	41.7 (39.4−44.0)
**Sex**				
Male	577	28.9 (25.7−32.2)	662	35.5 (31.3−39.7)
Female	1,124	32.3 (29.4−35.1)	1,410	44.2 (41.2−47.2)
**Age, y**				
18−24	42	24.5 (17.3−31.8)	74	43.7 (35.4−52.0)
25−34	135	36.0 (29.5−42.5)	201	54.3 (47.7−60.9)
35−44	239	33.2 (28.1−38.3)	317	48.4 (42.9−53.8)
45−54	442	35.9 (32.0−39.7)	496	40.3 (36.3−44.3)
≥55	843	26.1 (24.0−28.2)	984	29.9 (27.7−32.1)
**Education**				
Less than high school diploma	83	33.2 (24.6−41.9)	119	55.0 (45.9−64.1)
High school graduate	549	30.5 (26.5−34.6)	719	42.0 (37.7−46.3)
Some college	543	31.8 (28.0−35.6)	638	42.4 (38.3−46.5)
College graduate	526	28.7 (25.2−32.2)	596	36.9 (32.9−40.8)

Abbreviation: CI, confidence interval.

a Physical, sexual, or verbal abuse.

b Mental illness in a household member, substance abuse in a household member, witnessing abuse, divorce, or incarceration of a household member.

**Table 2:** Prevalence of Individual ACE Exposures and Prevalence of Each Individual ACE Category Within Direct^a^ and Environmental^b^ ACE Categories Among Non-Hispanic White Adults in Nebraska, Behavioral Risk Factor Surveillance System, 2011

ACE Exposure Category	No. of Respondents	% (95% CI)	Prevalence of Exposure Within ACE Exposure Category^c^
**Direct**			
Physical abuse	683	13.8 (12.2−15.4)	45.0 (40.8−49.3)
Sexual abuse	514	7.7 (6.7−8.8)	25.3 (22.0−28.6)
Verbal abuse	1,279	25.1 (23.2−27.2)	81.9 (79.1−84.7)
**Environmental**			
Mental illness in a household member	701	15.5 (13.6−17.3)	37.3 (33.5−41.1)
Substance abuse in a household member	1,172	23.6 (21.5−25.6)	56.7 (52.9−60.5)
Witnessing abuse	653	12.6 (11.0−14.1)	30.5 (27.0−34.0)
Incarceration of a household member	158	5.9 (4.5−7.2)	14.1 (11.1−17.2)
Divorce	734	19.2 (17.2−21.2)	46.1 (42.3−50.0)

Abbreviation: ACE, adverse childhood experience; CI, confidence interval.

a Physical, sexual, or verbal abuse.

b Mental illness in a household member, substance abuse in a household member, witnessing abuse, divorce, or incarceration of a household member.

c Proportions do not sum to 100% because persons might have more than 1 individual ACE within direct or environmental categories.

The prevalence of smoking by ACE exposure ranged from 22.2% (direct ACE) to 45.5% (both direct and environmental ACEs) (Table 3). After adjusting for age, sex, education level, and alcohol consumption, direct and environmental ACEs alone and in combination were significantly associated with smoking, with aRRs of 1.5, 1.8, and 2.7 for direct ACEs, environmental ACEs, and both categories, respectively.

**Table 3:** Prevalence and Adjusted Relative Risk of Smoking by ACE Exposure Among Non-Hispanic White Adults in Nebraska, Behavioral Risk Factor Surveillance System, 2011

ACE Exposure	n	Smoking, % (95% CI)	aRR^a^ (95% CI)
None	426	14.2 (11.9−16.5)	Reference
Direct^b^ only	113	22.2 (16.3−28.1)	1.5 (1.1−2.1)
Environmental^c^ only	243	30.3 (25.2−35.5)	1.8 (1.4−2.3)
Both	340	45.5 (40.1−50.9)	2.7 (2.2−3.3)

Abbreviation: ACE, adverse childhood experience; aRR, adjusted relative risk; CI, confidence interval.

a Adjusted for age, sex, education, and alcohol consumption.

b Physical, sexual, or verbal abuse.

c Mental illness in a household member, substance abuse in a household member, witnessing abuse, divorce, or incarceration of a household member.

## Discussion

Our results demonstrate that ACEs are common among Nebraska residents and that the categories of direct and environmental ACEs are both associated with an increased risk for smoking. These results are consistent with previous studies. ACEs have been linked to multiple health behaviors, including smoking (10,16), illicit drug use (10,17,18), alcohol misuse (10,19), obesity (10,20), and adolescent pregnancy (21). Dose-response relationships have been observed between increasing numbers of reported ACEs and smoking persistence among persons with tobacco-related illness (22).

We observed that a higher proportion of residents reported environmental rather than direct ACEs. More environmental than direct ACE categories exist; thus, the potential for experiencing environmental ACEs is likely greater. We demonstrated similar increases in risk for smoking with either direct or environmental ACEs alone. These results suggest that given the higher prevalence of environmental ACEs and its association with increased smoking risk, greater emphasis on preventing environmental ACEs in conjunction with preventing child abuse might decrease smoking among adults.

We evaluated ACEs by using a new method, aggregating individual ACEs into direct and environmental categories. Our method allowed us to compare risk of smoking among persons with direct ACEs, environmental ACEs, and both ACE categories, to persons without any ACEs. This method improves on previous studies comparing outcomes among persons who experienced a specific individual ACE with those of persons without that specific individual ACE, which could potentially diminish the association of the ACE and outcome because persons in the referent group could have ACEs other than the specific ACE under evaluation. For example, persons not experiencing physical abuse could have another individual ACE, such as household mental illness, that is associated with the outcome. Furthermore, comparing outcomes among persons with a specific individual ACE to those of persons without that specific individual ACE limits the ability to compare magnitude of association between 2 individual ACEs (in this example, physical abuse and household mental illness) and the outcome of interest because of differences in referent groups.

Our results using the new method could be useful as a state health department decides the most appropriate strategy to educate the general public about this issue. Explaining 2 categories and demonstrating associations between these categories and adverse outcomes could be useful in improving public knowledge without requiring in-depth explanations of each individual ACE.

ACEs can be considered early health determinants within the framework of LCT. To minimize the effects of ACEs on unhealthy behaviors and negative health outcomes later in life, LCT recommends primary prevention of ACEs through varied strategies aimed at providing the necessary supports throughout childhood development. At the same time, intervention on behalf of children at risk is important, highlighting the need for real-time, actionable surveillance data for each ACE category. Although states have mechanisms to detect and treat child abuse, the full burden of children residing in homes with mental illness, domestic violence, substance abuse, or incarcerated family members is unknown. As a Patient Protection and Affordable Care Act grantee for evidence-based home visiting programs, Nebraska’s Maternal and Child Health program is developing indicators for maternal and child well-being and determining appropriate data requirements to establish these indicators. A home visitation program was started during 2012, with the objective of preventing child abuse and neglect, and our study can be used to establish beneficial data collection for this program. Although little evidence exists that nurse home visits directly decrease child abuse and neglect, home visits can improve parenting practices and a child’s home environment (23–25). Efforts to improve child well-being in Nebraska can be fostered by providing the state with prevalence estimates of each ACE and with measures of association between ACEs and risk factors for disease. Nebraska stakeholders will be able to develop and modify plans for surveillance and intervention accordingly. These findings can also be used as an example for other states that have received a similar grant and will need to develop their own metrics.

Limitations of this study include the inability to verify self-reported history of ACEs and smoking. Studies evaluating accuracy of responses to questions in adulthood regarding childhood maltreatment demonstrate substantial underreporting but little overreporting (26,27). Thus, we likely provide conservative estimates of the association between smoking and ACEs among Nebraska residents. With a response rate of approximately 66%, differences between responders and nonresponders can introduce bias. Although BRFSS began incorporating cellular telephones into their survey methodology during 2011, the ACE module did not include cellular telephones, potentially underestimating ACE prevalence and associations between ACEs and smoking among persons aged 18–34 years. Finally, our sample did not include a sufficient number of persons of minority race/ethnicity to allow a comparison among different racial/ethnic groups in Nebraska. Strengths of this study include the use of predicted margins in our logistic regression model, enabling us to obtain relative risks rather than odds ratios. Because prevalence of both exposures and outcome were high, odds ratios do not provide an accurate approximation of relative risk. Sample size is another strength of this study, with more than 6,000 subjects in the final data set, which increases the power to detect associations.

ACEs are common in Nebraska and are associated with smoking, a risk factor that contributes to costly and disabling diseases. ACEs can be placed within the context of LCT to strategize methods that promote healthy conditions among all persons regardless of social, economic, and environmental circumstances. Surveillance systems to monitor a substantial number of childhood adverse events do not yet exist. State-specific strategies to monitor adverse events among children and provide interventions might help to decrease smoking among adults.

## Appendix. Behavioral Risk Factor Surveillance System (BRFSS) Questions and Responses Corresponding to 8 Adverse Childhood Experience (ACE) Categories

**Table:**

ACE Category	BRFSS Question	Response
Physical abuse	“How often did a parent or adult in your home ever hit, beat, kick, or physically hurt you in any way? Do not include spanking.”	“Once” or “More than once”

Sexual abuse	1. “How often did anyone at least five years older than you or an adult ever touch you sexually?”	“Once” or “More than once” to any question
	2. “How often did anyone at least five years older than you or an adult try to make you touch them sexually?”	
	3. “How often did anyone at least five years older than you or an adult force you to have sex?”	

Verbal abuse	“How often did a parent or adult in your home ever swear at you, insult you, or put you down?”	“More than once”

Mental illness in a household member	“Did you live with anyone who was depressed, mentally ill, or suicidal?”	“Yes”

Substance abuse in a household member	1. “Did you live with anyone who used illegal street drugs or who abused prescription medications?”	“Yes” to either question
	2. “Did you live with anyone who was a problem drinker or alcoholic?”	

Divorce	“Were your parents separated or divorced?”	“Yes”

Witnessed abuse	“How often did your parents or adults in your home ever slap, hit, kick, punch, or beat each other up?”	“Once” or “More than once”

Incarceration of a household member	“Did you live with anyone who served time or was sentenced to serve time in a prison, jail, or other correctional facility?”	“Yes”
